# TRPC1 Regulates the Activity of a Voltage-Dependent Nonselective Cation Current in Hippocampal CA1 Neurons

**DOI:** 10.3390/cells9020459

**Published:** 2020-02-18

**Authors:** Frauke Kepura, Eva Braun, Alexander Dietrich, Tim D. Plant

**Affiliations:** 1 Pharmakologisches Institut, BPC-Marburg, Fachbereich Medizin, Philipps-Universität Marburg, Karl-von-Frisch-Straße 2, 35043 Marburg, Germany; Frauke.Dormann@hs-gm.de (F.K.); braune@staff.uni-marburg.de (E.B.); alexander.dietrich@lrz.uni-muenchen.de (A.D.); 2 Walther-Straub-Institut für Pharmakologie und Toxikologie, Ludwig-Maximilians-Universität München, 80336 München, Germany; 3 Center for Mind, Brain and Behavior, Philipps-Universität Marburg, 35032 Marburg, Germany

**Keywords:** TRPC channels, cation channels, TRPC channel regulation, molecular physiology, TRPC1, nonselective cation current, hippocampus, CA1

## Abstract

The cation channel subunit TRPC1 is strongly expressed in central neurons including neurons in the CA1 region of the hippocampus where it forms complexes with TRPC4 and TRPC5. To investigate the functional role of TRPC1 in these neurons and in channel function, we compared current responses to group I metabotropic glutamate receptor (mGluR I) activation and looked for major differences in dendritic morphology in neurons from *TRPC1^+/+^* and *TRPC1^−/−^* mice. mGluR I stimulation resulted in the activation of a voltage-dependent nonselective cation current in both genotypes. Deletion of TRPC1 resulted in a modification of the shape of the current-voltage relationship, leading to an inward current increase. In current clamp recordings, the percentage of neurons that responded to depolarization in the presence of an mGluR I agonist with a plateau potential was increased in *TRPC1^−/−^* mice. There was also a small increase in the minor population of CA1 neurons that have more than one apical dendrite in *TRPC1^−/−^* mice. We conclude that TRPC1 has an inhibitory effect on receptor-operated nonselective cation channels in hippocampal CA1 neurons probably as a result of heterotetramer formation with other TRPC isoforms, and that TRPC1 deletion has only minor effects on dendritic morphology.

## 1. Introduction

Many neuron types respond to the activation of phospholipase C (PLC)-coupled metabotropic neurotransmitter or growth factor receptors with the activation of cation currents. Molecular candidates for some of the channels involved in these responses are members of the **c**anonical/**c**lassical TRP**C** subfamily of cation channels [1,2].

The seven TRPC isoforms (TRPC1–TRPC7) can be subdivided on the basis of sequence similarity and some functional properties into four groups (1: TRPC1, 2: TRPC2, 3: TRPC3/6/7 and 4: TRPC4/5) [3,4]. In the rodent brain, TRPC2 is mainly found in the vomeronasal organ, whereas the other TRPC channels are more widely expressed, with more than one isoform often being detected in the same region [4]. Since most cell types contain multiple TRPC isoforms, the formation of heterotetrameric channels with different properties is possible. All TRPC isoforms coassemble to form homomultimers, but TRPC1 is unlikely to form functional homomultimeric channels in the plasma membrane [5,6,7]. Group 3 and group 4 TRPCs can form heterotetrameric channels within their groups. There is also much evidence that TRPC1 can heteromultimerize with the more closely related isoforms in group 4 and some evidence for an interaction with group 3 TRPCs. However, in the adult brain, TRPC1 forms complexes with TRPC4 and TRPC5, but not with group 3 isoforms [8,9,10]. Heterologous co-expression of TRPC1 with TRPC4 or TRPC5 leads to channels with strongly modified functional properties compared to TRPC4 and TRPC5 homomers [5,7,11,12,13]. These include changes in the shape of the current-voltage (IV) relationship and a reduction in Ca^2+^ permeability.

In hippocampal CA1 neurons, an overlapping expression pattern [5,14,15,16,17] may lead to coassembly of TRPC1 with TRPC4 and TRPC5, and, in this and other regions of the rodent brain, metabotropic glutamate (mGluR) and muscarinic acetylcholine (mAChR) receptors activate TRP-like, Ca^2+^-dependent, nonselective cation conductances [18,19,20]. As well as possibly being involved in synaptic signaling in response to classical neurotransmitters, TRPC1 and TRPC5 have been reported to play a role in neuronal development and to influence neurite growth and growth cone turning and morphology [21,22,23,24,25,26]. Very recently, group 1 and group 4 TRPCs have been shown to regulate neurotransmitter release and short-term plasticity at glutamatergic synapses in the hippocampus independently of glutamate receptors by means of Ca^2+^-dependent activation [27].

To examine the role of TRPC1 in hippocampal CA1 neurons, we characterized the nonselective cation current activated by stimulation of group I mGluRs (mGluR I: mGluR1 and mGluR5) and studied neuron morphology and compared wild type mice (*TRPC1^+/+^*) with those lacking TRPC1 (*TRPC1^−/−^*). We show that mGluR I-activated, voltage-dependent, nonselective cation currents are increased in neurons from *TRPC1^−/−^* mice and that neuron morphology displays only modest but significant changes following deletion of TRPC1. The increase in inward current in *TRPC1^−/−^* neurons leads to an increase in the percentage of neurons that respond to depolarization in the presence of mGluR I agonists with a plateau potential.

## 2. Materials and Methods

### 2.1. Preparation of Hippocampal Tissue

Experiments were performed on hippocampi from male C57bl6/129SV wild type (*TRPC1^+/+^*) and homozygous knockout (*TRPC1^−/−^*) mice of postnatal ages between 10 and 35 days (P10–P35). The experiments involving animals were approved by the local council on animal care.

Mice were anaesthetized deeply with isoflurane (Baxter, Unterschleißheim, Germany) and then decapitated. The brain was quickly removed and immersed in an ice-cold, sucrose-containing cutting solution containing (in mM): 87 NaCl, 25 NaHCO_3_, 25 glucose, 75 sucrose, 2.5 KCl, 1.25 NaH_2_PO_4_, 0.5 CaCl_2_ and 7 MgCl_2_, equilibrated with 95% O_2_ and 5% CO_2_ [28]. One hemisphere was used for making slices, and, in some experiments, the hippocampus from the second hemisphere was used for RNA isolation. In some cases, both hippocampi were used for RNA isolation or rapid Golgi staining (see below).

### 2.2. Slice Preparation, Patch-Clamp Recordings and Data Analysis

Thin horizontal hippocampal slices (200 µm) were prepared using a vibratome slicer (HR2, Sigmann-Elektronik, Hüffenhardt, Germany) while bathed in ice-cold cutting solution. After cutting, slices were maintained submerged in a chamber filled with gassed artificial cerebrospinal fluid (ACSF) containing (in mM): 126 NaCl, 25 NaHCO_3_, 10 glucose, 2.5 KCl, 1.4 NaH_2_PO_4_, 2 CaCl_2_, 4 MgCl_2_ and 1.3 ascorbic acid, and allowed to recover for 30 min at 35 °C before being used for experiments. Slices were kept at room temperature for up to 8 h.

After the recovery period, slices were transferred to a submerged recording chamber and superfused continuously (2.5–3.5 mL/min at room temperature) with ACSF containing (in mM): 126 NaCl, 25 NaHCO_3_, 10 glucose, 2.5 CsCl (for voltage-clamp experiments) or 2.5 KCl (for current-clamp experiments), 1.4 NaH_2_PO_4_, 2 CaCl_2_ and 1 MgCl_2_, equilibrated with 95% O_2_ and 5% CO_2_.

Patch pipettes were pulled from borosilicate glass (Science Products, Hofheim, Germany) and filled with an internal solution containing (in mM): 110 Cs-methanesulfonate, 25 CsCl, 30 HEPES, 1 EGTA, 0.362 CaCl_2_, 2 MgCl_2_, pH 7.2 with CsOH (290–300 mOsm) with a calculated free [Ca^2+^] of 100 nM for voltage-clamp recordings. For current-clamp recordings, patch pipettes were filled with an internal solution containing (in mM): 135 K-gluconate, 5 KCl, 10 HEPES, 0.1 EGTA, 3 MgCl_2_, pH 7.3 with KOH (290–300 mOsm). Patch pipettes had resistances of 3–5.5 MΩ when filled with the intracellular solution.

In all experiments, fast excitatory and inhibitory synaptic transmission was blocked by 10 µM 6,7-dinitroquinoxaline-2,3-dione disodium salt (DNQX,), 25 µM d-(−)-2-amino-5-phosphonopentanoic acid (D-AP5) and 200 µM picrotoxin (all from BioTrend, Cologne, Germany). In some experiments, 500 nM tetrodotoxin (TTX, Alomone Labs, Jerusalem, Israel) was added to the superfusate solution to block voltage-gated sodium channels. G_q_-coupled metabotropic glutamate receptors type I (mGluR I) were stimulated by bath application of 100 µM (RS)-3,5-dihydroxyphenylglycine (DHPG, BioTrend).

Whole-cell patch-clamp recordings were obtained from CA1 hippocampal neurons visualized by infrared differential interference contrast (IR-DIC) video microscopy with a IR CCD camera (VX55, TILL Photonics, Gräfelfing, Germany) mounted on an upright microscope (BX51WI, Olympus, Hamburg, Germany). Recordings in current-clamp and voltage-clamp modes were made using an EPC-10 patch-clamp amplifier (HEKA Elektronik, Lambrecht/Pfalz, Germany).

Experiments with uncompensated series resistances ≤25 MΩ were accepted in this study and 40% of the series resistance was compensated electronically. Data were acquired using PULSE (HEKA Elektronik). In voltage-clamp experiments, cells were held at a potential of −60 mV. Current-voltage (IV) relationships were obtained from voltage ramps with a duration of 400 ms applied at 0.1 Hz. Three different types of ramp were used: The standard ramp protocol was a voltage step from the holding potential (−60 mV) to +60 mV for 50 ms followed by a ramp to −100 mV. To test the voltage dependence of the current, we used either a “triangular” ramp from −100 mV to +60 mV and after 50 ms a ramp back to −100 mV, or voltage steps to different potentials between −50 to +60 mV at which the neuron was held for 50 ms followed by a ramp to −100 mV. In the latter, to reduce the effect of current decay during agonist application, ramps were applied at a higher frequency of 0.4 Hz and only five potential steps including a step to +60 mV were tested in each experiment. In all experiments, with the exception of Figure 3e, the ramps reflect the largest response recorded during agonist application. Ramp data were acquired with a sampling rate of 2.0 kHz after filtering at 0.4 kHz.

In current-clamp experiments, the effect of depolarizing current pulses (+20 pA, 2 s) before and during DHPG application was investigated in neurons kept close to −60 mV by current injection. Data were digitized at 5 kHz after filtering at 1.7 kHz.

Analysis and plotting were carried out using IgorPro 6.03 (WaveMetrics, Lake Oswego, OR, USA), and GraphPad Prism4 or 5 (GraphPad Software, San Diego, CA, USA). Data in Figure 3e were fitted to a Boltzmann sigmoid (GraphPad Prism5):

I_-60_ (normalized) = 1/1 + e^−(V − V½)/slope^ (1)

where V_½_ is the potential of half-maximal activation. From the slope the valence (z) was calculated as −25/slope, assuming a temperature of 20 °C.

### 2.3. Preparation of Whole Hippocampi, Reverse-Transcription Polymerase Chain Reaction Analysis and Quantitative Real-Time PCR

For RNA isolation and quantitative real-time PCR (qRT-PCR) experiments, the hippocampus was isolated as described previously [29].

Total RNA from hippocampal tissue was isolated using TRI Reagent (Sigma-Aldrich, Taufkirchen, Germany). First strand synthesis was carried out with random hexamer primers (Thermo Fischer Scientific, Darmstadt, Germany), using RevertAid™ M-MuLV Reverse Transcriptase (Thermo Fischer Scientific). PCR reactions were carried out using the following conditions: initial denaturation for 5 min at 70 °C, 15 min at 25 °C, 60 min at 42 °C followed by a final extension at 70 °C for 10 min. Samples were stored at −20 °C until use.

After reverse transcription, specific fragments from the cDNAs for TRPC1, TRPC3, TRPC4, TRPC5, TRPC6, TRPC7 and β-actin, which served as “housekeeping gene”, were simultaneously amplified using primer pairs described previously (TRPC1, [30]; TRPC3–7, [31]

Real-time PCR was performed using the ABsolute™ QPCR SYBR^®^ Green mix (Thermo Fischer Scientific) containing a Thermo-Start^®^ DNA Polymerase, reaction buffer, nucleotides and active SYBR^®^ Green I dye. 2 µL of each primer pair and 2 μL from the first strand synthesis (as a 1:10 dilution) were added to the reaction mixture, and PCR was carried out in a LightCycler^®^ (Roche, Mannheim, Germany) using the following conditions: 15 min initial activation and 45 cycles of 12 s at 95 °C, 30 s at 55 °C, 30 s at 72 °C and 10 s at 81 °C each [30]. Fluorescence intensities were recorded after the extension step at 81 °C after each cycle to exclude fluorescence of primer dimers melting lower than 80 °C. Samples containing primer dimers were excluded by melting curve analysis. Crossing points were determined by the software. The relative gene expression was quantified using the formula:

(2e^(Crossing point^^β-actin − Crossing point X)^) × 100 = percent of reference gene expression (2)

To investigate the presence and size of the amplified fragments, PCR products were separated by electrophoresis and visualized in ethidium bromide-stained agarose gels (2%; see Supplementary Figure S1).

### 2.4. Genotyping of Mice

The genotype of the mice used was verified with PCR using primers pairs and conditions described previously followed by agarose gel electrophoresis [30].

### 2.5. Golgi Impregnation and Image Analysis

For rapid Golgi staining, brains from mice of postnatal age 20–25 d were dissected from the skull and the hemispheres were placed in fixative containing: 5 g potassium dichromate, 5 g chloral hydrate, 8 mL glutaraldehyde, 6 mL formaldehyde, 10 drops DMSO diluted in 100 mL ddH_2_O [32]. The tissue blocks were kept in the fixative for six days in the dark at room temperature, and the fixative changed every second day. Seven days after preparation, tissue blocks were washed several times in 0.75% silver nitrate solution and stored in this solution in the dark at room temperature for another four days. After impregnation, tissue blocks were transferred to 30% sucrose for three days. Horizontal slices (thickness: 120 µm) were cut using a vibratome (HR2, Sigmann-Elektronik) and collected in 6% sucrose [33]. Slices were then sequentially dehydrated in ethanol (50%, 70%, 90%, for 2 min each, then twice for 5 min in 100%), cleared with xylene for 15 min, mounted on glass slides (Superfrost Plus, Menzel, Braunschweig, Germany) and covered with Canada balsam. Finally, the slides were air-dried in the dark.

Impregnated sections from five independent mouse brains of each genotype (*TRPC1^+/+^* and *TRPC1^−/−^*) were imaged and analyzed through a 40x water objective. First, the number of primary branches from 59–134 neurons per brain was counted. Thereafter, the apical dendrites of CA1 hippocampal neurons were classified into three categories simplified after Nakamura et al. [34]: (I) neurons with a single apical trunk; (II) neurons with trunks that bifurcated at the border of *stratum pyramidale* and *stratum radiatum* or in the *stratum radiatum*; and (III) neurons that possess two or more apical dendrites. From each mouse brain, 112–248 neurons were analyzed. The percentage of each category was scored and averaged in each genotype.

### 2.6. Statistical Analysis

All values are reported as means ± SEM. Statistical significance was tested using Student’s t test, a Mann–Whitney test, a one-way ANOVA or a two-way ANOVA. The criterion for significance was *p* < 0.05.

## 3. Results

### 3.1. Group I mGluR-Activated cation Currents are Increased in Hippocampal CA1 Neurons from TRPC1^−/−^ Mice

**Figure 1:**
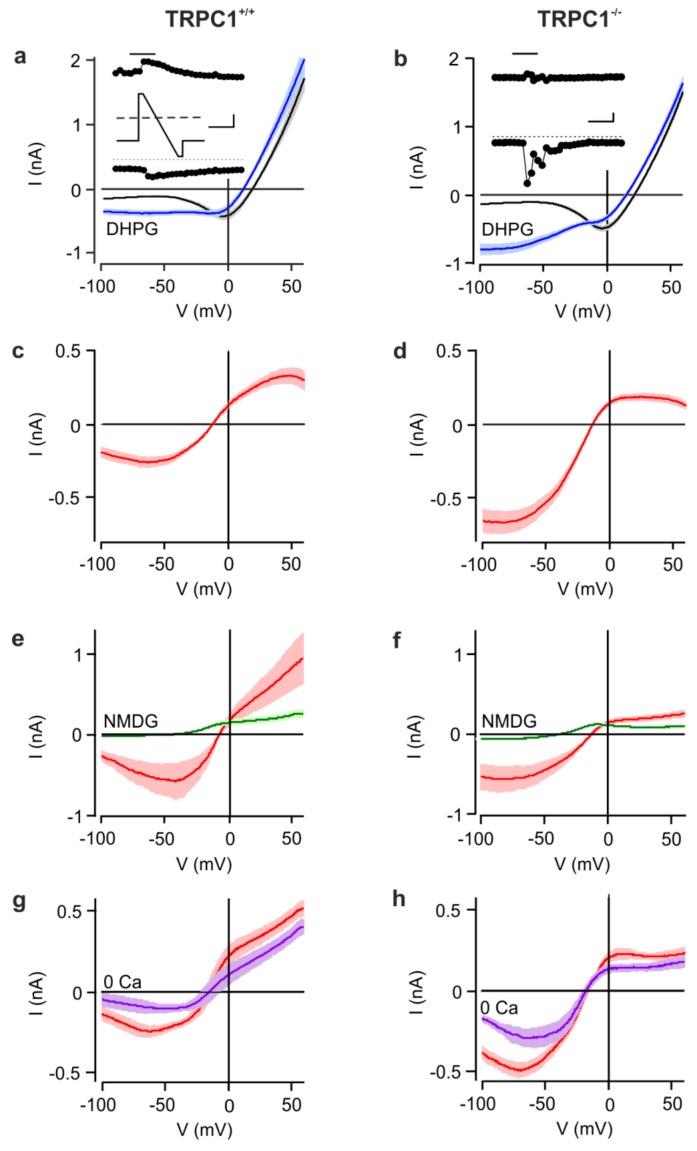
mGluR I-activated cation currents in CA1 neurons from *TRPC1^+/+^* and *TRPC1^−/−^* mice. (**a**,**b**) Mean current-voltage (IV) relationships of *TRPC1^+/+^* (a) and *TRPC1^−/−^* neurons (b) obtained from voltage ramps from +60 to −100 mV (see inset to (a)) before (black) and during (blue) activation of mGluR I by DHPG (100 µM). The insets (filled circles) show the time courses of currents at −100 mV (lower trace) and +60 mV (upper trace) during a representative experiment. The bar above the upper trace indicates the time at which DHPG was applied. The scale bars represent 0.25 nA and 60 s. IVs are means ± SEM with *n* = 13 (*TRPC1^+/+^*) and *n* = 25 (*TRPC1^−/−^*). Data from 14 to 26-day-old mice. (**c**,**d**) DHPG-activated currents (red) in *TRPC1^+/+^* (c) and *TRPC1^−/−^* neurons (d). The data are from the same experiments as (a,b). (**e**,**f**) DHPG-activated currents in *TRPC1^+/+^* (e) and *TRPC1^−/−^* neurons (f) in a HEPES-buffered control (red, *n* = 5 and 7, respectively) and in a Na^+^- and Ca^2+^-free (*N*-methyl-d-glucamine (NMDG)) solution (green, *n* = 6 and 5 respectively). (**g**,**h**) DHPG-activated currents in *TRPC1^+/+^* (g) and *TRPC1^−/−^* neurons (h)))) in a control solution (red, *n* = 6 and 12, respectively) and in a nominally Ca^2+^-free solution (0 Ca, purple, *n* = 7 and 4, respectively).

Because TRPC channel subunits form receptor-operated cation channels, we compared mGluR I-activated cation currents in whole-cell voltage-clamp recordings from CA1 neurons in horizontal hippocampal slices from 14- to 26-day-old *TRPC1^+/+^* and *TRPC1^−/−^* mice (Figure 1). Neurons were held at −60 mV and current responses to voltage ramps from +60 to -100 mV recorded before and in response to a 1 min application of the mGluR I agonist (RS)-3,5-dihydroxyphenylglycine (DHPG, 100 µM). To reduce contamination by other conductances, recordings were made in the presence of inhibitors of ionotropic glutamate receptors and GABA_A_ receptors, and K^+^ was replaced by Cs^+^ to reduce K^+^ currents.

**Figure 2:**
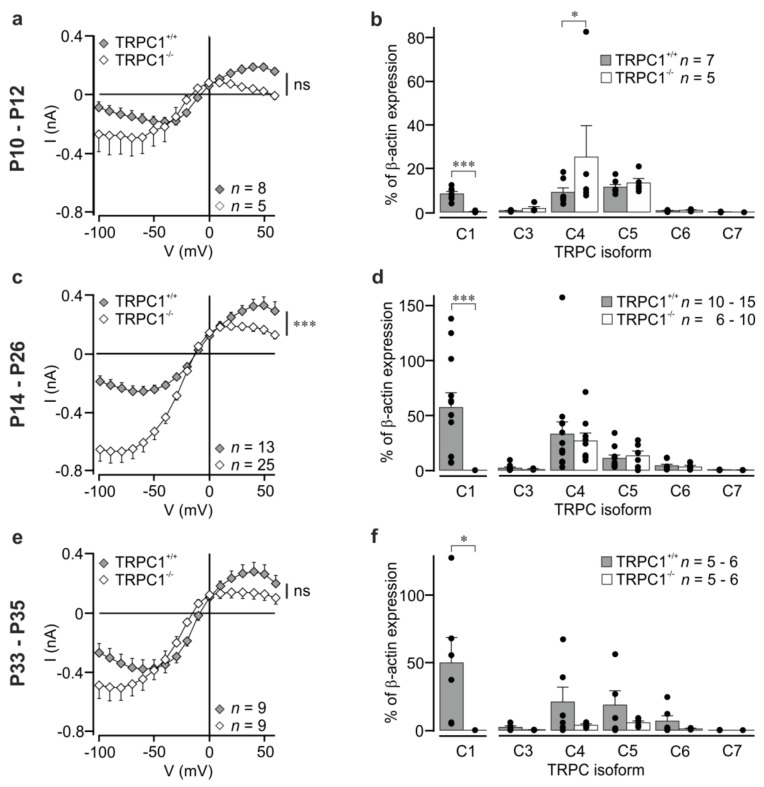
Mean mGluR I-activated currents and TRPC isoform expression at three different stages of postnatal development. (**a**,**c**,**e**) Mean DHPG-activated current-voltage relationships in CA1 neurons from *TRPC1^+/+^* (filled symbols) and *TRPC1^−/−^* mice (open symbols) in the age ranges P10–P12, P14–P26, and P33–P35, respectively. Values are means ± SEM. The asterisks at the side indicate the result of a two-way ANOVA. The data in (c) are the same data as those shown in Figure 1c,d. All points in (a,c,e) were extracted from ramp data at 10 mV intervals. (**b**,**d**,**f**) TRPC isoform expression determined by qRT-PCR in the hippocampi of *TRPC1^+/+^* (filled columns) and *TRPC1^−/−^* mice (open columns) in the age ranges P10–P12, P14–P26, and P33–P35, respectively. The dots show the individual values, and the number of experiments per genotype is shown in each panel. Asterisks indicate: *** *p* < 0.001; * *p* < 0.05, and ns not significant (Student’s t test for TRPC1; two-way ANOVA with Bonferroni post-hoc test for TRPC3–TRPC7).

Before DHPG application, currents from both genotypes were not significantly different at any potential (Figure 1 a,b). During the application of DHPG, inward and outward currents transiently increased and the reversal potential (V_rev_) shifted to more negative potentials. The most striking difference between the genotypes was the larger increase in inward current in *TRPC1^−/−^* neurons at negative membrane potentials (Figure 1b), resulting in currents that at −60 mV were, on average, about twice as large as in *TRPC1^+/+^* neurons (Figure 1a,b). In *TRPC1^+/+^* neurons, the current-voltage (IV) relationship of the DHPG-activated current was S-shaped with a minimum around −50 mV, a maximum at +40 mV and a V_rev_ around −10 mV (Figure 1c and Figure 2c). In contrast, the IV-relationship from *TRPC1^−/−^* neurons had a reduced region of negative slope at potentials more negative than −50 mV, or lacked this region entirely, showed smaller outward currents, but had a similar V_rev_ (Figure 1d and Figure 2c). The differences in the IV-relationships were statistically significant (two-way ANOVA of values in Figure 2c: *p* < 0.001 and individual values at potentials negative to −40 mV (Bonferroni post hoc test).

In neurons of both genotypes, DHPG activated only small inward currents in a Na^+^-, Cs^+^- and Ca^2+^-free extracellular solution (NMDG^+^; Figure 1e,f), and V_rev_ was shifted strongly to more negative potentials, indicating that the inward current is largely carried by cations. In a nominally Ca^2+^-free, Na^+^-containing extracellular solution (0 mM Ca^2+^), mean DHPG-sensitive currents in *TRPC1^+/+^* and *TRPC1^−/−^* neurons were smaller than those in Ca^2+^ (Figure 1g,h), but the reduction in *TRPC1^−/−^* neurons was not significant (Figure 1h). V_rev_ was unaffected by Ca^2+^ removal.

We also measured currents in neurons from younger animals (P10–P12) prior to eye-opening, and in older animals (P33–P35) to see whether the differences were maintained in other age groups. In general, the shape of the IV was changed in all groups, but, owing to the large differences in current amplitude in individual neurons, current differenceswere not statistically significant (Figure 2a,e; two-way ANOVA with Bonferroni post-hoc test). A parameter that is independent of current amplitudes is the relationship between the absolute current amplitudes at -60 mV and at +40 mV. These values were around 1 for *TRPC1^+/+^* neurons (P10–P12: 0.89 ± 0.18; P14–P26: 0.87 ± 0.1; P33–P35: 1.58 ± 0.23) and larger than 1 for *TRPC1^−/−^* neurons (P10–P12: 14.41 ± 5.72; P14–P26: 4.90 ± 0.77; P33–P35: 6.17 ± 1.91) with statistically significant differences between the genotypes (*p* < 0.05 at P10–P12 and P33–P35, and *p* < 0.001 at P14–P26; Mann–Whitney test). The result reflects the stronger inward rectification in *TRPC1^−/−^* neurons.

### 3.2. TRPC Channel Expression in Hippocampi from TRPC1^+/+^ and TRPC1^−/−^ Mice

Since genetic deletion of a TRPC channel can result in changes in the expression levels of other TRPC isoforms and lead to increases in currents [35], we quantified TRPC mRNA levels in total RNA from whole hippocampi from *TRPC1^−/−^* and *TRPC1^+/+^* mice using qRT-PCR. Levels of TRPCs, except TRPC2, which is only expressed in the VNO [4], were quantified relative to β-actin. The sequence of the TRPC1 forward primer is localized in the region of exon 8, which is deleted in *TRPC1^−/−^* mice, and can distinguish between *TRPC1^−/−^* and *TRPC1^+/+^* mice [30]. As expected, mRNA for TRPC1 was not detected in *TRPC1^−/−^* mice (Figure 2b,d,f). Deletion of TRPC1 did not have a statistically significant effect on the expression of other TRPC isoforms (TRPC3–TRPC7; Figure 2b,d,f), with the exception of TRPC4 at P10–P12 and this is owing to a single high value (Figure 2b). A statistically significant increase in TRPC1 mRNA was measured between P10–P12 and P14–P16 in *TRPC1^+/+^* mice (*p* <0.05, one-way ANOVA with Tukey post-hoc test), and an increase in TRPC3 mRNA between P10–P12 and P33–P35 in *TRPC1^−/−^* mice (*p* < 0.05, one-way ANOVA with Tukey post-hoc test). No other effects of age were observed.

### 3.3. The Group I mGluR-Activated Cation Current Modified by TRPC1 is Voltage-Dependent

**Figure 3:**
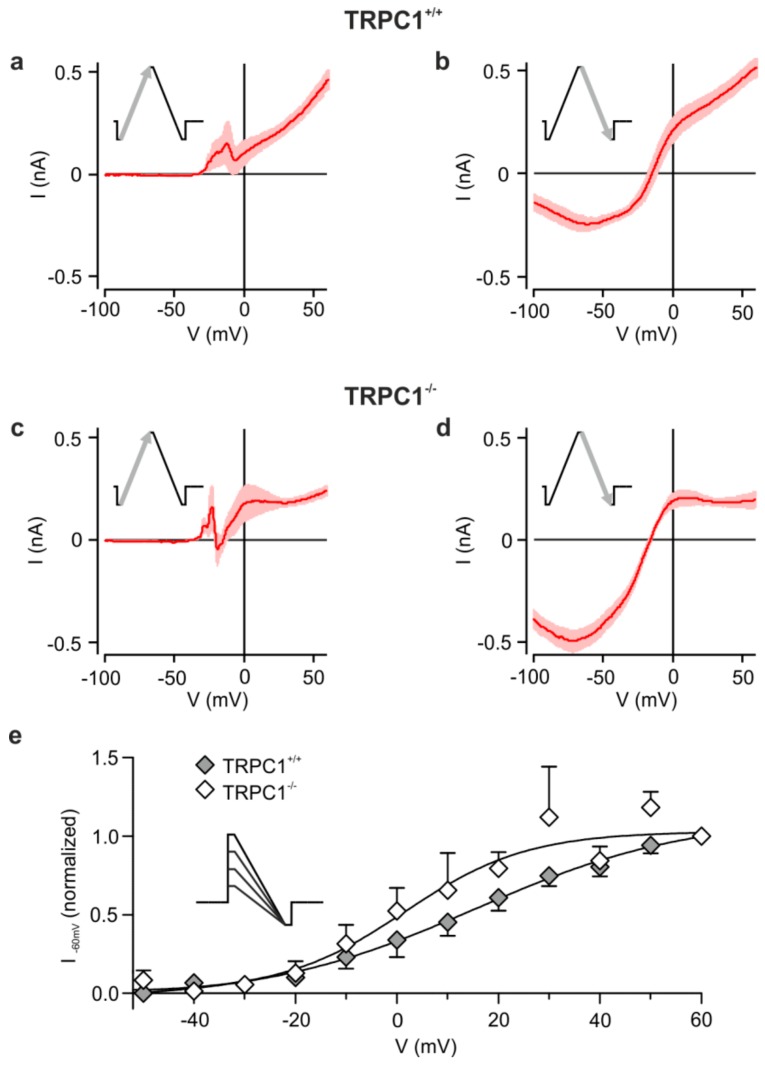
Voltage dependence of mGluR I-activated currents. (**a**,**c**) Mean IV relationships of the DHPG-activated current obtained from voltage ramps from −100 to +60 mV in *TRPC1^+/+^* and *TRPC1^−/−^* neurons. (**b**,**d**) Response from the same neurons as in (a, c) to a ramp from +60 to −100 mV. Ramp responses were recorded in TTX. Results are means ± SEM with *n* = 6 (*TRPC1^+/+^*) and *n* = 10 (*TRPC1^−/−^*). (**e**) Effect of step potential on the DHPG-sensitive current at −60 mV. Currents at −60 mV were measured during ramps to −100 mV from different potentials (see inset), normalized to the current following a step to +60 mV and plotted against the initial potential for *TRPC1^+/+^* (filled symbols) and *TRPC1^−/−^* (open symbols). Values are the means of 4–18 experiments, with the exception of the values at −50 mV for *TRPC1^+/+^* (*n* = 2) and at +50 mV for *TRPC1^−/−^* (*n* = 3). The continuous curves are fits of the average data to a Boltzmann equation (Equation 1) with respective V_½_ and z values of 15.5 mV and 1.2 for *TRPC1^+/+^* and 1.5 mV and 2.1 for *TRPC1^−/−^*.

As shown in Figure 3 with triangular ramps from −100 to +60 mV and back, we found that the DHPG-activated inward currents in *TRPC1^+/+^* and *TRPC1^−/−^* neurons depend on the direction of the ramp. Outward, but little inward current, was seen during the ascending phase (Figure 3a,c), but a strong inward component was observed during the descending phase (Figure 3b,d). Our results suggest that depolarization and DHPG are necessary to activate the conductance.

Because tail currents after voltage steps were difficult to measure, we attempted to estimate the voltage dependence by applying voltage steps to different potentials (−50 to +60 mV) followed by a ramp to −100 mV (inset to Figure 3) and measured the current at -60 mV. We applied only a few ramps, always including a step to +60 mV, above which currents did not increase, to each neuron.

The averaged data normalized to the +60 mV response show a voltage dependence (Figure 3e) like that seen for ascending ramps (Figure 3a). Currents increased between −30 mV and +50 mV, with half-maximal activation between 0 and +20 mV in neurons from *TRPC1^+/+^* and *TRPC1^−/−^* mice. Genotype did not have a statistically significant effect on the relationships between activation and potential at any potential (two-way ANOVA with Bonferroni post-hoc test). However, fits to the average data hinted that half maximal activation may occur at more negative potentials in neurons from *TRPC1^−/−^* mice. Thus, the DHPG-sensitive current in both genotypes is weakly voltage dependent and may activate at slightly more negative potentials in *TRPC1^−/−^* mice.

### 3.4. TRPC1^−/−^ Neurons Show More Plateau Potentials in Response to Current Injection in the Presence of an mGluR I Agonist

To investigate the effect of the increased DHPG-sensitive inward current in CA1 neurons from TRPC1^−/−^ mice on the membrane potential, we performed whole-cell current-clamp recordings in the presence of inhibitors of ionotropic glutamate and GABA_A_ receptors before and after application of 100 µM DHPG in TRPC1^+/+^ and TRPC1^−/−^ mice.

**Figure 4:**
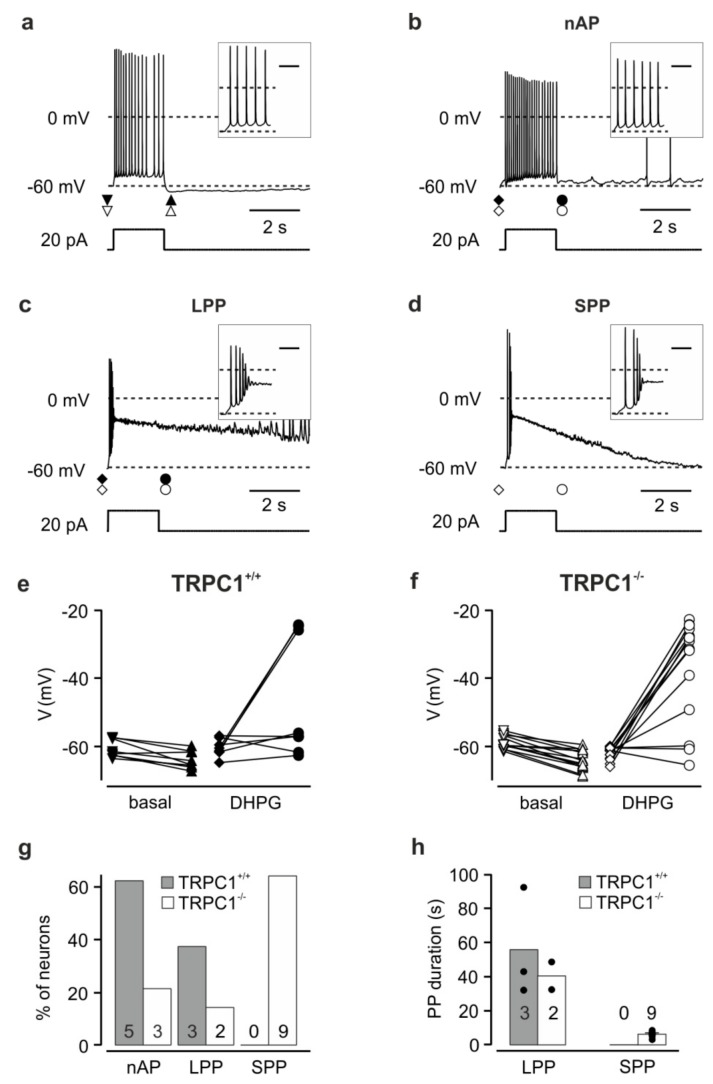
Effects of depolarizing current injections on the membrane potential of *TRPC1^+/+^* and *TRPC1^−/−^* neurons. (**a**) Response typical for neurons of both genotypes before DHPG application. Data from a *TRPC1^−/−^* neuron. Current injection: +20 pA. (**b**–**d**) Examples of activity patterns observed in response to current injection in the presence of DHPG. (**b**) A *TRPC1^+/+^* neuron that showed no afterpotential (nAP). (**c**) A *TRPC1^+/+^* neuron that showed a long plateau potential (LPP). (**d**) A *TRPC1^−/−^* neuron that showed a short plateau potential (SPP). The insets show the beginning of the reponse on an expanded time scale. The scale bar in the inset represents 0.2 s. Neurons were kept at potentials close to −60 mV by current injection. The symbols under the potential traces in (a–d) indicate the times at which the potentials in (e, f) were measured**.** (**e**, **f**) Potential before and after the depolarizing current injection in the absence (basal) and presence of DHPG (DHPG) in *TRPC1^+/+^* (e) and *TRPC1^−/−^* (f) neurons. (**g**) Percentage of cells showing each response type in DHPG. (**h**) Duration of plateau potentials in response to current injection in DHPG in *TRPC1^+/+^* (filled bars) and *TRPC1^−/−^* neurons (open bars). Numbers on the columns in (g, h) indicate the number of neurons. Dots indicate the values from individual neurons.

TRPC1^+/+^ and TRPC1^−/−^ neurons had similar mean membrane potentials under these conditions of −60.4 ± 1.4 mV (*n* = 16) and -63.5 ± 1.0 mV (*n* = 23; *p* = 0.073), respectively. In all CA1 neurons from both TRPC1^+/+^ and TRPC1^−/−^ mice in the absence of DHPG, current injection (+20 pA, 2 s) to neurons at a potential close to −60 mV induced a small depolarization and repetitive action potential firing (Figure 4a; *n* = 8 and 14, respectively). After current injection, most neurons showed a weak afterhyperpolarization (Figure 4e,f; ΔV TRPC1^+/+^: −3.3 ± 0.8 mV, *n* = 8; TRPC1^−/−^: −5.3 ± 0.6 mV, *n* = 14, *p* = 0.0673). In contrast, during the application of DHPG, the same current injection resulted in two different reaction patterns in TRPC1^+/+^ neurons. In most neurons (62.5%; Figure 4g), an action potential train was elicited during the current injection. Subsequently, the membrane potential returned to a value near that before the depolarization (no afterpotential: nAP, Figure 4b,e; ΔV: +1.1 ± 1.6 mV, *n* = 5). The remaining three neurons (37.5%; Figure 4g) responded with an initial depolarization and action potentials followed by a second stronger depolarization with high frequency firing and a depolarization block of action potential activity (Figure 4c). The second depolarization (maximum: −9.6 ± 9.2 mV, *n* = 3) formed a slowly-declining plateau potential (PP) during the current injection (potential immediately after the 2 s current injection: −25.0 ± 0.5 mV, *n* = 3) and persisted for several tens of seconds before repolarizing (long plateau potential, LPP, Figure 4c). The duration of the depolarization (plateau and repolarization) was between 33.4 and 92.3 s (mean: 55.8 ± 18.5 s, *n* = 3, Figure 4h). The repolarization with superimposed action potentials was followed by an afterhyperpolarization (not seen on the timescale in Figure 4c). In some *TRPC1^−/−^* neurons, DHPG induced the same reaction patterns described for *TRPC1^+/+^* (Figure 4f,g): a small depolarization with repetitive firing (nAP; 23.1%) or an LPP (15.4%). However, in most neurons (61.5%), a rapid depolarization with a few action potentials preceded a shorter PP (SPP, Figure 4d,g,h), not observed in *TRPC1^+/+^* neurons (Figure 4g). This response resembled the LPP in amplitude (Figure 4c,d,f; LPP to −16.1 mV, *n* = 2, SPP to −22.7 ± 3.5 mV, *n* = 9), but repolarized more rapidly (Figure 4d,h; SPP: 3.2–8.6 s, mean: 6.3 ± 0.5 s, *n* = 9, LPP: 32.4 and 48.6 s). Like the LPP, the SPP was followed by an afterhyperpolarization. In summary, *TRPC1^−/−^* neurons are more likely to generate PPs in response to depolarization in the presence of an mGluR I agonist than *TRPC1^+/+^* neurons, but the pleateau is mostly shorter.

### 3.5. Morphology of Hippocampal Neurons from TRPC1^+/+^ and TRPC1^−/−^ Mice

**Figure 5:**
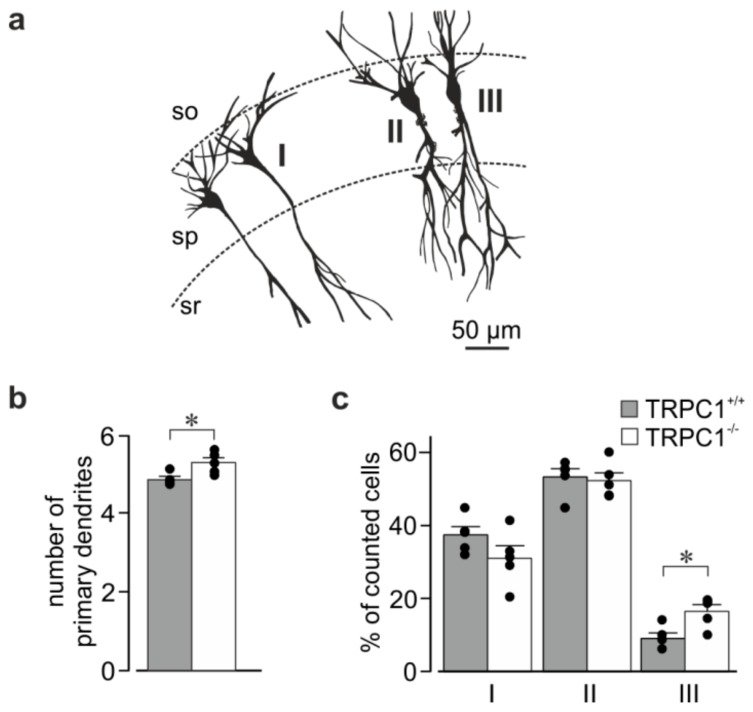
Major dendrite morphology in CA1 neurons from *TRPC1^+/+^* and *TRPC1^−/−^* mice. (**a**) Tracings of four Golgi-stained neurons in one slice showing two neurons of category I (left), and single neurons of categories II and III (right) into which neurons from *TRPC1^+/+^* and *TRPC1^−/−^* mice were classified. SO: *stratum oriens*, SP: *stratum pyramidale*, SM: *stratum moleculare*. (**b**) The average number of primary dendrites (sum of apical and basal dendrites) in CA1 neurons from *TRPC1^+/+^* and *TRPC1^−/−^* mice. (**c**) Percentage of neurons showing the branching patterns I, II and III (see (a)) in *TRPC1^+/+^* and *TRPC1^−/−^* mice. The data are means ± SEM from five mice of each genotype and 112–248 neurons per mouse, and * indicates *p* < 0.05, Student’s t test). Dots indicate the values from individual mice.

*TRPC1^−/−^* mice have no obvious phenotype [30], and the size and appearance of the hippocampus was similar in slices from mice of both genotypes. To see if deletion of TRPC1 had an effect on the major branching pattern of CA1 neurons, we looked at the morphology of neurons in rapid Golgi-stained sections (Figure 5). CA1 neurons from *TRPC1^−/−^* mice had slightly more primary dendrites (sum of basal and apical dendrites) than *TRPC1^+/+^* neurons (Figure 5b). The apical dendrite morphology differed slightly between the genotypes with the minor population of neurons having two apical dendrites (type III) being increased in *TRPC1^−/−^* mice (Figure 5a,c). In both genotypes, most neurons had dendrites that bifurcated at the border of the *stratum pyramidale* and *stratum radiatum* or in the *stratum radiatum* (type II), followed by those with a single apical trunk (type I).

## 4. Discussion

The principal findings of this study are that deletion of TRPC1 modifies a receptor-operated Ca^2+^-dependent, nonselective cation current leading to stronger inward rectification and larger inward currents in CA1 neurons. Deletion also increases the number of neurons that respond to depolarization in the presence of mGluR I agonists with PPs.

Larger cation currents are unlikely to be explained by an increase in the expression of other TRPC subunits, as observed in vascular smooth muscle with TRPC3 following deletion of TRPC6 [35], because deletion of TRPC1 does not affect mRNA levels for nearly all TRPC subunits in the hippocampus of our animals, or in adult animals in another TRPC1 knockout [36]. The only significant increase, which may be responsible for differences in currents at this age, was in TRPC4 mRNA at P10–P12, but no increase was seen in the other age groups. A limitation of this study is that we only measured mRNA levels and, owing to the lack of quantitative data at the protein level, we cannot rule out that changes in the protein expression of other subunits may be responsible for the changes seen in *TRPC1^−/−^* neurons. An increase rather than a decrease in current indicates that the receptor-operated cation channel in CA1 neurons is not formed by TRPC1 homomultimers. The IV relationships in *TRPC1^+/+^* and *TRPC1^−/−^* CA1 neurons resemble those seen after coexpression of TRPC1 with TRPC4 or TRPC5, and homomeric TRPC4 or TRPC5, respectively [5,7,11,12,13], and these isoforms are most abundant in neurons in the CA1 region [5,14,15,16]. Consistent with other studies that showed a reduction in inward current through other TRPC isoforms when co-expressed with TRPC1 [5,7,11,12,13], we could show that TRPC1 deletion in native cells leads to an increase in inward current. In addition to the shape of the IV-relationship, the dependence of the neuronal current on extracellular Ca^2+^ is also consistent with a contribution of group 4 TRPC channels [5,37,38,39]. Although the formation of heteromultimers with other TRPC subunits is a likely explanation for our findings, we cannot exclude the possibility that TRPC1 modifies the activity of an endogenous non-TRPC cation channel or that TRPC1 changes Ca^2+^ handling by the neurons.

A striking difference between the CA1 neuron cation conductance and that of heterologously-expressed hetero- or homomeric TRPC combinations is the absence of current at negative potentials without a prior depolarization. TRPC channels have been reported to be voltage-dependent [40,41], but, except at very low levels of activation, usually show an inward current without membrane depolarization [42]. The voltage dependence of the current in CA1 neurons and its steepness are not consistent with an activation or potentiation by cation (e.g., Na^+^ or Ca^2+^) entry through voltage-gated channels, as currents persist in the nominal absence of extracellular Ca^2+^, and in TTX. The voltage dependence and difference in IV shape in *TRPC1^−/−^* and *TRPC1^+/+^* neurons, that leads to larger currents could also be explained by models of voltage-dependent activation of TRP channels from other subfamilies where a weak intrinsic voltage dependence of the channel shifts from very positive potentials into the physiological range during the course of activation [43]. We did not observe a clear statistically significant shift in voltage dependence to more negative potentials in *TRPC1^−/−^* neurons. However, any shift may be obscured because the potential dependence also varied strongly between neurons of one genotype. The variation is not surprising given that activation is voltage dependent and that the voltage dependence, which is controlled by multiple factors, likely shifts along the voltage axis during the activation/inactivation process [40,41]. A reduction in single channel conductance, as observed in HEK293 or CHO cells for heteromeric TRPC combinations [5,7], and which cannot be explained by a shift in the voltage dependence, is also a possible explanation for the current reduction in the presence of TRPC1. However, our attempts to measure unitary DHPG-activated currents in CA1 neurons were unsuccessful (data not shown). A further possibility to explain the difference in voltage dependence between native and heterologously expressed channels is that channel properties in neurons are modified by interaction partners not expressed in cells used for expression. Deletion of TRPC1 should increase the proportion of homo- and heteromeric group 4 TRPCs. In addition to changing the IV shape, this should also lead to an increase in Ca^2+^ permeability of the remaining channels [12], leading to stronger Ca^2+^ entry and Ca^2+^ dependent activation of TRPC4 and TRPC5 containing channels [38,39].

The properties of the TRPC1-regulated current are consistent with a role in the generation of PPs. Following depolarization (and action potentials) in the presence of an agonist at G_q_-coupled receptors, the cation channel will contribute an inward current which depolarizes the membrane to potentials slightly more negative than 0 mV. The increase in this current in *TRPC1^−/−^* neurons could explain the higher percentage of neurons that generate PPs. The shorter duration of the plateau in *TRPC1^−/−^* neurons may be a result of the more rapid turn-off of this or other depolarizing currents in this genotype, or the stronger activation of repolarizing conductances secondary to activation of the larger cation current and increases in Ca^2+^ entry. Similar PPs occur in response to mAChR activation in CA1 neurons, and have been shown to involve cation channels and high voltage-activated Ca^2+^ channels [44]. Upregulation of Ca_V_2.3 Ca^2+^ channels [45] and inhibition of slow K^+^ channels could also contribute to PPs in CA1 neurons [46]. In addition, other studies on afterdepolarizations and currents in response to mGluR or mAChR activation in hippocampal CA3 and cortical neurons show involvement of a voltage-dependent cation conductance [47,48].

Our results suggesting an involvement of TRPC1 in the generation of PPs are consistent with other studies showing that TRPC channels, and in particular group 4 TRPC channels, with which TRPC1 can heteromultimerize, contribute to PPs or afterdepolarizations in response to G_q_-coupled receptor activation in both hippocampal, septal and cortical neurons [49,50,51,52,53]. The role of TRPC1 that our data support in CA1 neurons contrasts with one of these studies that also used *TRPC1^−/−^* animals and showed that septal neurons from mice lacking TRPC1 and TRPC4 did not generate PPs, and those from *TRPC1^−/−^* mice developed fewer PPs in response to mGluR activation and depolarization [53]. However, the PPs in septal neurons were much shorter than those that we observe, but were recorded at higher temperatures. Another study that contrasts with ours showed that TRPC1 contributes to the mGluR-induced increase in action potential frequency in CA1 neurons, but this relied on the specificity of antibodies used to inhibit TRPC isoforms [52]. Other studies on animals lacking TRPC isoforms have shown that deletion of TRPC5 reduces G protein-regulated cation currents in neurons of the amygdala [54], and that deletion of TRPC4 or TRPC5 reduces mGluR I-excitatory postsynaptic currents in the same region [17,54]. Deletion of TRPC3 abolishes mGluR1-induced depolarization and cation currents in cerebellar Purkinje neurons [31]. In the latter, deletion of TRPC1 and TRPC4 had no effect on the response. TRPC1 has also recently been reported to contribute with TRPC4 to a long-lasting depolarization in olfactory bulb granule cells, again shorter than that that we describe, that is dependent on NMDA receptor activation [55].

Recent studies have addressed the role of TRPC channels in hippocampal function. Triple knockout animals lacking TRPC1, TRPC4 and TRPC5 show deficits in spatial working memory and relearning competence [10]. Here, the TRPC channel complex affected glutamate release from excitatory inputs, but did not affect postsynaptic function including long-term potentiation (LTP) or depotentiation [10]. In this case, the Ca^2+^-activated TRPC channel isoforms [38,39] are activated by the presynaptic Ca^2+^ increase rather than by mGluRs and, owing to their Ca^2+^ permeability, contribute to the rise in intracellular Ca^2+^ [27]. Thus, TRPCs regulate transmitter release and short-term plasticity. *TRPC1^−/−^* animals from a different mouse strain to that we used also showed deficits in spatial working memory and fear conditioning [36]. This study found a decrease in excitability at CA3 to CA1 synapses and a reduction in LTP and long-term depression in brain slices, and a reduction in mGluR I-induced Ca^2+^ entry in cultured hippocampal neurons from *TRPC1^−/−^* mice [36]. Although not directly comparable, our results would predict a postsynaptic effect and increased mGluR I mediated responses in neurons from *TRPC1^−/−^* animals. A presynaptic effect would not have been observed under our experimental conditions.

In addition to a role in responses to G_q_-coupled receptors, TRPC4, TRPC5 and TRPC1, have been reported to be involved in responses to growth factors, and to be important for neurite growth, growth cone turning and morphology, and neuronal migration [11,12,21,22,23,24,25,26]. Gross hippocampal morphology was unchanged in *TRPC1^−/−^* mice. However, a closer look revealed an increase in the mean number of primary dendrites in CA1 neurons. This resulted from an increase in neurons with two apical dendrites (type III), while the number of neurons with only a single apical dendrite with no major branches (type I) was decreased, although not significantly. Since the population of neurons with two apical dendrites is still very small in *TRPC1^−/−^* mice (16.5% vs. 9% in *TRPC1^+/+^*), the physiological consequence of this change may be difficult to detect. However small, this effect suggests an inhibitory action of TRPC1 on dendritic outgrowth from the soma; a process that occurs prenatally. Deletion of TRPC5 also affects dendritic morphology in CA1 neurons by increasing the number of primary dendrites, dendritic length and branching points [56]. In cultured hippocampal neurons, transfection of TRPC4 or TRPC5 reduced neurite length and branching, whereas knockdown of endogenous TRPC4 had the opposite effect [26].

## 5. Conclusions

In conclusion, the nonselective cation current regulated by TRPC1 in CA1 neurons requires both the activation of mGluR I together with membrane depolarization for activation. In this respect, like the NMDA receptor, it acts as a coincidence detector for membrane depolarization and glutamate release. TRPC1 reduces mGluR I-activated currents and excitability by decreasing the tendency to generate PPs. In addition, our data indicate that lack of TRPC1 has a minor effect on dendritic architecture.

## Supplementary Materials

The following are available online at https://www.mdpi.com/2073-4409/9/2/459/s1, Supplementary Figure S1: qRT-PCR products from *TRPC1^+/+^* and *TRPC1^-/-^* mice.
