# Supplementary material for: TRPC1 Regulates the Activity of a Voltage-Dependent Nonselective Cation Current in Hippocampal CA1 Neurons
Source: Cells. 2020 Feb 18;9(2):459. doi: 10.3390/cells9020459 (PMC7072794; doi:10.3390/cells9020459)
Supplement: Supplementary file 1 [file cells-09-00459-s001.pdf]

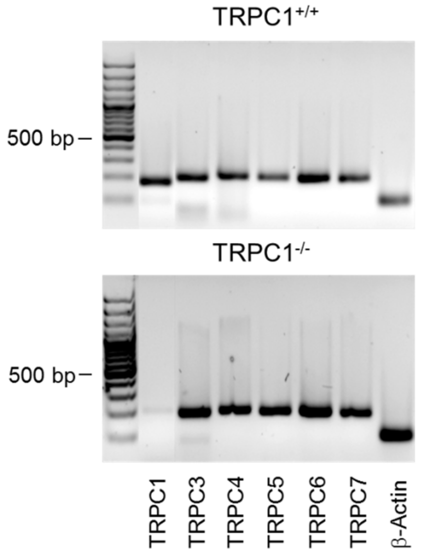

Supplementary Figure S1. qRT-PCR products from *TRPC1*$^{+/+}$ and *TRPC1*$^{-/-}$ mice. Agarose gels of qRT-PCR products for TRPC1 and TRPC3 - 7 from the hippocampi of 25- and 18-day-old *TRPC1*$^{+/+}$ and *TRPC1*$^{-/-}$ mice respectively. The expected fragment sizes were TRPC1: 179bp; TRPC3: 207bp; TRPC4: 216 bp; TRPC5: 210 bp; TRPC6: 209 bp; TRPC7: 205 bp; β-actin: 106 bp. The ladder is a 100 bp ladder (Thermo Fischer Scientific).
